# *In vitro* preliminary study of osteoblast response to surface roughness of titanium discs and topical application of melatonin

**DOI:** 10.4317/medoral.19953

**Published:** 2014-08-17

**Authors:** Maria-Fernanda Solá-Ruiz, Carolina Pérez-Martínez, José-Javier Martín-del-Llano, Carmen Carda-Batalla, Carlos Labaig-Rueda

**Affiliations:** 1 Associate Lecturer in Prosthodontics and Occlusion. Faculty of Medicine and Dentistry, Department of Dental Medicine, University of Valencia, Spain; 2 Master of Dental Prosthetics. University of Valencia, Spain; 3 Senior Lecturer in Histology. Faculty of Medicine and Dentistry, Department of Pathology, University of Valencia, Spain; 4 Professor of Histology. Faculty of Medicine and Dentistry, Department of Pathology, University of Valencia, Spain; 5 Senior Lecturer in Prosthodontics and Occlusion, Faculty of Medicine and Dentistry, Department of Dental Medicine, University of Valencia, Spain

## Abstract

Objectives: To observe human osteoblast behavior cultured *in vitro* on titanium discs (Ti) in relation to surface roughness and melatonin application. Study Design: Human osteoblasts (MG-63) were cultured on 60 Ti6Al4V discs divided into three groups: Group I: discs treated with dual acid etching; Group II dual acid etching and blasting with calcium phosphate particles; Group III (control) machined discs. Surface roughness and topography of the discs were examined with scanning electron microscope (SEM) and confocal laser scanning electron microscope( CLSM). Osteoblast adhesion, proliferation and cell morphology were determined by means of fluorescence microscopy with Image-Pro Plus software and SEM. Results: Group II presented the roughest discs, while the least rough were Group III. Cell adhesion was greatest in Group II. The addition of melatonin improved cell proliferation. Conclusions: 1. Surface treatments (dual acid etching, calcium phosphate impaction) increase surface roughness in comparison with machined titanium. 2. Greater surface roughness tends to favor cell adhesion after 24-hour cell culture. 3. The addition of melatonin tends to favor osteoblast proliferation.

** Key words:**Osteoblasts, titanium, roughness, melatonin, dental implants, osseointegration.

## Introduction

The clinical success of dental implants depends on osseointegration, the direct structural and functional connection between living bone and the implant surface. The osseointegration process is initiated by osteoblast precursor cells, which actively migrate towards the implant surface producing osteogenic factors that are capable of inducing the progressive differentiation of these cells into osteoblasts. Implant surface modifications have an important effect on this process and the subsequent development of bone tissue (1).

Successful osseointegration, both in the short- and long-term, depends on multiple factors, among which, implant surface characteristics (physicochemical and topographic) are of key importance (2).

Surface topography (roughness and texture) can be considered a very influential surface property for conditioning the response of the organism to the presence of the implant and has been studied both *in vitro* (3-7) and *in vivo* (2,8-10). Various studies point out that when the surface roughness of commercially pure titanium is incremented (Ti c.p.) beyond the roughness produced by machining, this will improve osteoblast response *in vitro* (3-7) and osseointegration *in vivo*. As roughness increases, so does the quantity of bone in contact with the implant and the implant’s resistance to loosening (2,8-10).

One surface treatment in common use is acid etching, which consists of bathing the titanium implant in an acid – hydrochloric acid, sulphuric acid, nitric acid or hydrofluoric acid - so that a uniform micro texturing is produced over the titanium surface by the subtraction of material (without any residue), while the loss of metal from the implant body is kept under control. (11) Bathing can be in a single acid or a combination of acids (dual etching with two acids or triple etching with three); better results are found when different acids are combined (8-10,12,13).

Melatonin is a synthesized hormone secreted by the pineal gland and was applied topically to implants by Cutando *et al*. (14) it is considered one of the most powerful and complete antioxidants because of its capacity for eliminating, purifying or neutralizing free radicals.(14,15) In bone formation, melatonin stimulates the proliferation and synthesis of type I collagen in human osteoblasts *in vitro* (16).

In preosteoblast cultures, it has been observed that melatonin increases the gene expression of bone sialoprotein and other bone protein markers, including alkaline phosphotase (ALP) and osteocalcin, dose-dependently accelerating cell differentiation (17,18). It has been shown that, due to melatonin`s antioxidant capacity, it can interfere and inhibit bone resorption by inhibiting osteoclast activity by means of Receptor Activator of Nuclear factor-Kappa B (RANK), as it reduces the messenger ribonucleic acid (mRNA) expression of this type I membrane protein on the surface of osteoclasts, so inhibiting osteoclastogenesis (19). In this way, melatonin could help dental implant osseointegration due to both its stimulating effect on bone formation and its inhibiting effect on bone resorption.

For these reasons the present study postulated the following null hypotheses:

1st - Surface treatment influences titanium’s surface roughness (Ra).

2nd - Increasing roughness favors osteoblast cell growth.

3rd - The addition of melatonin favors osteoblast cell growth.

The study objectives were.

1. To examine and quantify surface topography and roughness of titanium discs generated by different surface treatments, prior to cell culture.

2. To observe the influence of the roughness of the treated titanium surfaces on adhesion, proliferation and morphology of human osteoblasts cultured *in vitro*.

3. To observe the influence of melatonin application on adhesion, proliferation and morphology of human osteoblasts cultured *in vitro*.

## Study Design

The study used 60 discs sized 10 x 2 mm. manufactured by Biomet 3i® (Paterna, Spain) made from an alloy of titanium, aluminum and vanadium (Ti6Al4V) used for fabricating dental implants. The discs were distributed randomly into three groups of twenty: Group I, discs treated with dual acid etching (hydrochloric and sulphuric acid); Group II, dual acid etching and blasting with calcium phosphate particles; Group III (control group), machined discs without any additional surface treatment.

Prior to cell culture, the discs were analyzed under an optical microscope (Carl Zeiss, OPMI Pico Dental, Germany) and scanning electron microscope (SEM) (Jeol JSM 6300; Oxford Instruments Ltd. Abingdon, U.K.).

Mean roughness (Ra) was quantified for each disc using a con focal laser scanning electron microscope (CSLM) (Lext OLS3100; Olympus Corporation, Tokyo, Japan). Three evaluations per disc were performed. Afterwards all discs were sterilized by gamma rays and packaged to keep them sterile for cell culture.

- Cell Culture 

The study used human osteoblasts from the cell line MG-63 (ATCC, reference CRL-1427). Cells were incubated at 37o in a 5% CO2/95% air atmosphere at 100% relative humidity, using DMEM as culture medium (Dulbecco’s modified Eagle Medium) containing 10% fetal bovine serum (inactivated by heat after treatment for 30 min at 56o), 2 mN glutamine, 1 mN sodium pyruvate, nonessential amino acids at a concentration of 1 x penicillin (100 units/mL) and streptomycin (100 µg/mL). The 60 disks were distributed in 24-well plates.

The cells were trypsinized, resuspended in the culture medium described above and seeded to a density of 1×104 cells/mL. To determine the effect of melatonin on cell interaction and growth, melatonin culture medium (Sigma-Aldrich, St. Louis, MO, U.S.A.) with a final concentration of 50 µM was added to half the Group I and Group II disks.

- Adhesion and cell proliferation 

**Figure 1:**
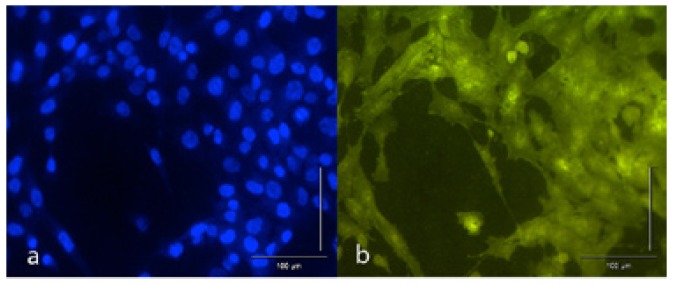
Image captured under fluorescence microscope (20x).(a) DAPI staining attaches to nuclei DNA allowing cells to be counted.(b) Eosin staining attaches to cell cytoplasm making it possible to observe different morphology patterns.

To assess adhesion and cell proliferation, after 24 and 72 hours (respectively) in culture, the disks were washed in the wells with phosphate buffered saline (PBS) three times, fixing the cells with glutaraldehyde 2.5% in PBS for 30 min at room temperature. After four washes in PBS, samples were stained sequentially with hematoxylin and eosin (5 min each wash, washing in abundant water between staining), and lastly incubated with 4',6-diamidino-2-phenylindole (DAPI) fluorophore dissolved in PBS for staining. Afterwards the disks were examined under a fluorescence microscope using the appropriate filters. Images were captured with 10x and 20x lenses to determine both the number of cells (counting nuclei whose DNA was stained with DAPI) and cell morphology (through fluorescence emission from the eosin attached to cells) (Fig. 1. a,b).

- Cell morphology 

Morphometric analysis of the images captured under the fluorescence microscope was performed using Image-Pro Plus software (version 6.0.0.260 Media Cybernetics, Rockville, MD 20850 USA).

Six disks (two per group) were prepared for SEM examination after 24 and 72 hours culture. The cells were lysed and kept in 100% ethanol to perform the ‘critical point’ process in order to dehydrate cells in a CO2 atmosphere in a controlled manner for perfect conservation of the cell morphology and condition. Lastly the disks were covered with a fine layer of gold.

- Statistical analysis

Statistical analysis was performed using the SPSS-Windows statistical package, applying the Kruskal-Wallis and Mann-Whitney tests. The statistical confidence level was set at 95% (α=0.05).

## Results

Mean roughness (Ra) analysis data obtained using CLSM identified the roughest disks in Group II, with a mean Ra of 0.316 µm, followed by Group I with a mean Ra of 0.277 µm and in last place Group III with a mean Ra of 0.114 µm.

Statistically significant differences were found between the three groups (Kruskal-Wallis test: *p*<0.001).

Images captured under optical microscopy and SEM revealed the irregular texture of the treated discs (Groups I and II) with pits and holes, without discernable differences between these two groups, while the machined surface of the control group (Group III) presented regular concentric machining lines.

After 24 hours in culture, osteoblasts actively adhered to the treated surfaces and the machined surface.

**Figure 2:**
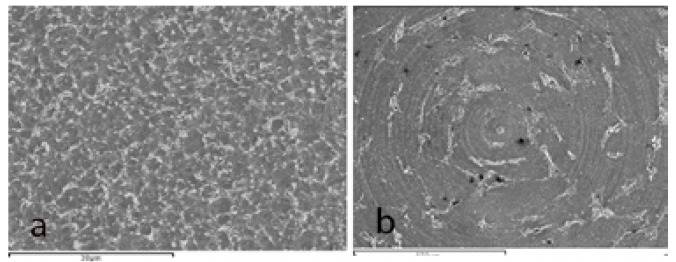
SEM microphotos (a) On treated disks (Group II) osteoblasts proliferated randomly over the surface. (b) On Group III machined disks, osteoblasts proliferated in lines following the lines generated by machining.

Microphotos captured by SEM showed differences between the treated surfaces and the machined surface and how these differences affected cell behavior. On treated disks (Groups I and II) osteoblasts proliferated randomly over the surface, while on Group III machined disks, osteoblasts proliferated in lines following the lines generated by machining (Fig. 2. a,b).

The analysis of cell adhesion after 24 hours in culture, performed by immunofluorescent techniques, identified the greatest osteoblast cell adhesion in Group II, corresponding to greater surface roughness. The addition of melatonin improved results obtained by Group I disks, but this did not occur in the same way for Group II disks ( Table 1).

**Table 1:**
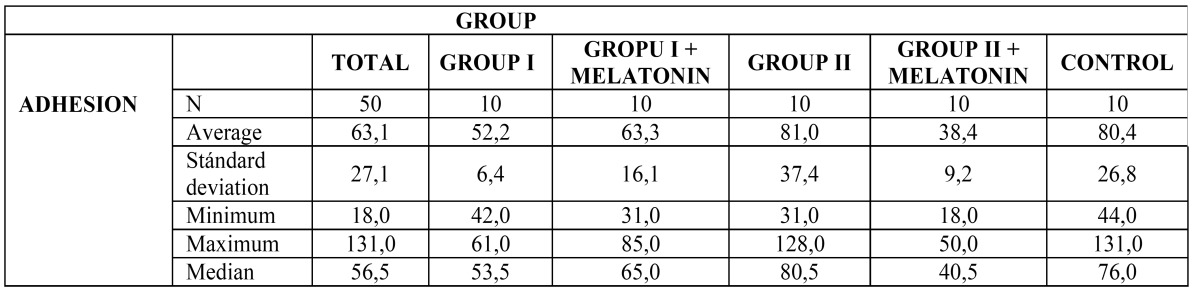
Descriptive results show cell adhesion after 24 hours in culture in all groups.

 Proliferation analysis after 72 hours in culture showed how the addition of melatonin improved results for both Group I and Group II disks, with Group I achieving the best results ( Table 2).

**Table 2:**
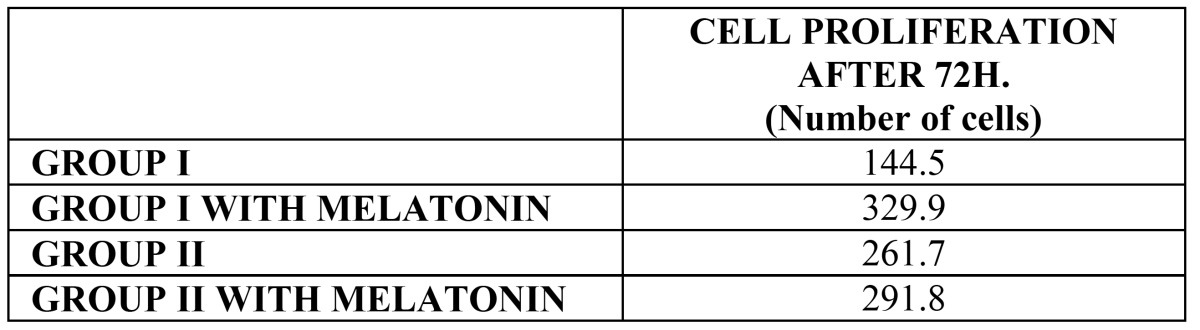
Table shows cell proliferation after 72 hours in culture of Group I and II, and with melatonin addition.

**Figure 3:**
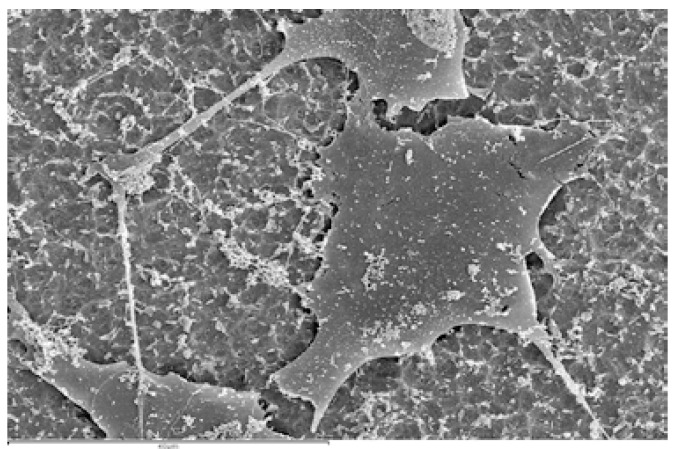
SEM microphotograph (1500x) of Group II disk (with its surface characteristics). Note the flattened morphology adopted by osteoblasts with cytoplasmic extensions and filopods in order to better adhere to the surface and spread over it.

Regarding cell morphology, osteoblasts were found to adapt to the titanium surface flattening themselves and propagating cytoplasmic extensions which helped cells to better adhere to the surface and to spread over the surface (Fig. 3).

However, not all cells were in this state: when cell division commenced, cells appraised the available space in their vicinity by means of intercellular signals, and gave up their flattened form, retracting the extensions, to divide and separate, followed by a return to the flattened form and the propagation of pseudopods.

## Discussion

This study set out to analyze the influence of micro topography and titanium surface roughness on osteoblast adhesion, proliferation and osteoblast morphology.

Short-term *in vitro* studies providing data obtained from cultivating osteoblasts on different substrata can be used to generate information about the first reaction events between implant and osteoprogenitor cells. They also help to identify and select the most advantageous substrata in terms of rapid healing and bone regeneration, and furthermore, interference in long-term responses (3,4). Various authors have validated *in vitro* studies involving osteoblasts on different substrata, including both the cells and culture media used in this study (3,4,7).

In spite of the generalized use of profilometry for surface characterization and for quantifying roughness, this method is limited in terms of resolution, and for this reason the present study used a confocal laser scanning electron microscope (7,20). This analysis obtained similar results to other researchers, with Ra values for machined disks of between 0.1 and 0.2 µm, while treated disks obtained higher values of between 0.2 and 0.3 µm, as in studies by Eisenbarth (5) and da Silva (6).

In this way, surface treatment does influence the roughness (Ra) of titanium and so the present study’s first hypothesis was validated.

It is known that cell adhesion to titanium surfaces is a key factor in successful implant osseointegration. Research has shown that titanium surface roughness is directly associated with osteoblast adhesion and proliferation and the consequent development of mineralized tissue at the implant interface (21,22).

The present study obtained better results for cell adhesion for rough surfaces, indicating that micro texturing favors cell accommodation, a finding corroborated by da Silva (6) and Pae (7). However, it should be noted that although the results indicated this tendency, it would be necessary to increase the sample size in order to reach any conclusive statistical power.

This study was conceived in the belief that the addition of melatonin acts as a mechanism for improving osteoblast response and osseointegration, on the basis of this substance’s capacity for stimulating bone formation (16-18) and for reducing resorption by inhibiting osteoclastogenesis (19). *In vivo* studies have shown that two to four weeks after local application around implants, melatonin boosts bone-to-implant contact (BIC), increases total peri-implant bone - and also inter-thread bone - and trabecular density while decreasing total peri-implant and inter-thread conjunctive tissue.(15,23) In the present study, it was found that the addition of melatonin improved results for cell proliferation. This agrees with Satomura (24), although Zhang (25) reported an initial increase in adhesion and proliferation but failed to find any difference at longer culture times.

It has not been possible to corroborate this study’s second and third hypotheses due to the small sample size, which needs to be enlarged in future research.

Regarding cell morphology, cytoplasmic extensions and filopodia were observed, together with a flattened morphology in close contact with the disk surface, phenomena also observed by da Silva (6), Pae (7) and Yamano (26) in their respective studies. It was not possible to identify clear differences in cell morphology between the two treated surfaces, although completely different proliferation patterns were observed between the treated titanium surfaces and the manufacturer’s machined surface, where cell proliferation followed a concentric pattern following the lines generated by machining, while on the treated surfaces, cells proliferated randomly over the surface.

Surface treatments (dual acid etching, calcium phosphate particle blasting) increase surface roughness in comparison with machined titanium.

Greater surface roughness tends to favor osteoblast adhesion after 24-hour cell culture.

The addition of melatonin tends to favor osteoblast proliferation after 72-hour cell culture.

Recognizing the limitations imposed by the small sample size, further *in vitro* research in this direction will require larger samples.
